# Perceived Nutrition and Health Concerns: Do They Protect against Unhealthy Dietary Patterns in Polish Adults?

**DOI:** 10.3390/nu13010170

**Published:** 2021-01-08

**Authors:** Małgorzata Ewa Drywień, Jadwiga Hamulka, Marzena Jezewska-Zychowicz

**Affiliations:** 1 Department of Human Nutrition, Institute of Human Nutrition Sciences, Warsaw University of Life Sciences (SGGW-WULS), Nowoursynowska 159C, 02-776 Warsaw, Poland; malgorzata_drywien@sggw.edu.pl (M.E.D.); jadwiga_hamulka@sggw.edu.pl (J.H.); 2 Department of Food Market and Consumer Research, Institute of Human Nutrition Sciences, Warsaw University of Life Sciences (SGGW-WULS), Nowoursynowska 159C, 02-776 Warsaw, Poland

**Keywords:** dietary patterns, discretionary foods, perception, concerns, health, nutrition, adults

## Abstract

The aim of the study was to explore the associations between perceived health and nutrition concerns, sociodemographic characteristics and unhealthy dietary patterns in a representative group of Polish adults. The data were collected in 2017 through a cross-sectional quantitative survey under the National Health Program 2016–2020. Logistic regression models were used to analyze the data. It was found that higher health concerns decreased the chances of adhering to upper tertiles of “Fast food & alcohol” and “Refined food & sweetened beverages” dietary patterns (DPs), thus displaying less frequent consumption of such foods. No relationship was found between health concerns and adhering to the “Fat food” and “Butter” DPs. Nutrition concerns increased the likelihood of frequent consumption of foods from “Fast food & alcohol” DP. Women were less likely to adhere to unhealthy eating patterns than men, while older people were less likely to often consume fast food, alcohol, or refined food and sweetened beverages. Findings of this study showed that concerns about health or nutrition were differently associated with dietary patterns and consumption of unhealthy foods. These relationships should be considered when developing interventions to address health-related lifestyle changes. However, further research is needed to identify cause-effect relationships between these variables.

## 1. Introduction

The public interest in health and nutrition is constantly increasing; however, at the same time, widespread occurrence of diet-related non-communicable diseases, especially obesity, type 2 diabetes mellitus, hypertension, coronary heart disease, and metabolic syndrome, is still observed [1,2,3]. An unhealthy diet, characterized by high sugar and fat intake and low intake of fruit and vegetables is one of the leading causes of non-communicable diseases globally [3] and is strongly associated with early mortality [4,5]. Simultaneously, interventions aimed at changing eating behaviors have demonstrated only modest effects over time [6]. Previous studies indicate clearly that gaining a better understanding of unhealthy diet predictors is necessary in order to increase effectiveness of actions focused on reducing the occurrence of undesirable behaviors [7,8]. To achieve this goal, it is reasonable to focus on dietary patterns that reveal the complex nature of diet by considering the synergistic effects of foods and nutrients within the body as well as the way we consume food [9,10]. Until now, such studies most often took into account particular groups of food products and predictors of their consumption [11,12]. Despite the widespread promotion of healthy lifestyle, the WHO recommendations have not been implemented sufficiently in the Polish population [13] nor in other European countries [14,15]. So-called discretionary foods and drinks, which are not needed to meet nutritional requirements but contribute to overall food satisfaction even if consumed occasionally or in small amounts, may cause deterioration of health [16]. Despite the fact that unhealthy food is consumed in excessive amounts [17,18] and the potential risks associated with it are often underestimated by consumers [19]. Thus, the perceived risk/concerns about health and nutrition should be considered as a factor of vital importance for understanding and then modifying undesirable behaviors. Until now, researchers have most often used two constructs to study this predictor of an individual’s behavior, i.e., “health interests” [20] or “health concern” [21,22], which reflect either the importance of or the concerns about health, nutrition, and food. Some research has been focused on understanding consumer perception of food risks, while other studies have taken into account risks and concerns connected to one’s own lifestyle including eating behaviors [23]. Regardless of the difference in approach to the perceived risk/concerns, it is considered that it tends to be lower than the actual risk [24,25]. However, the impact of perceived risk on behavioral change is still unclear. Several studies have found a positive relationship between perceived risk and health-related behaviors, while others have not [26,27]. This relationship should be further examined, especially regarding non-recommended foods (i.e., discretionary foods).

It can be expected that unhealthy dietary patterns are differentiated internally [28,29] and therefore an individual’s characteristics, including demographics and those related to health and nutrition concerns, may have different predictive importance. Thus, we hypothesized that those who had a high level of concerns about health or nutrition would display unhealthy dietary behaviors to a lesser extent. The rationale for this hypothesis was that a high level of concerns may be an important motive in establishing and maintaining more healthy dietary behaviors. Moreover, our assumption that unhealthy dietary patterns are differentiated internally allows formulating the hypothesis that the prediction of unhealthy dietary patterns differs in conformance with various sociodemographic characteristics of the group. To date, limited evidence has been reported on the associations between perceived health and nutrition concerns and dietary patterns, including unhealthy dietary patterns (DPs). Thus, the aim of the study was to explore the associations between perceived health and nutrition concerns, sociodemographic characteristics, and unhealthy dietary patterns in a representative group of Polish adults.

## 2. Materials and Methods

### 2.1. Ethical Approval

The study was approved by the Ethics Committee of the Faculty of Human Nutrition and Consumer Science, Warsaw University of Life Sciences, Poland, on 19 June 2017 (Resolution No. 22/2017). The study was conducted according to the guidelines laid down in the Declaration of Helsinki. Before starting the interview, the interviewer explained the purpose of the survey and asked the respondent for consent to participate. 

### 2.2. Study Design and Sample Collection

The data were collected in November–December 2017 through a cross-sectional quantitative survey under the National Health Program 2016–2020. According to the study design, recruitment and data collection were conducted by a research agency—KANTAR TNS. The computer-assisted personal interviewing (CAPI) technique was used to collect all data.

The sample for the study was a random, stratified representation of the total population of Poland aged 18–86 years old. The study group was recruited from Universal Electronic System of Population Register (PESEL) which is administered by the Polish Ministry of Digitization. The bases for stratification of the sample were data on the demographics of Poland, published by the Central Statistical Office in Poland (CSO-GUS), current as of 30 June 2016. Individuals were selected by the method of systematic drawing within distinguished strata. Stratification took into account the size of the city (9 classes) and their territorial distribution (16 provinces—voivodships). The required number of people was drawn, i.e., 1000 respondents and a 3-fold reserve for the sample. Therefore, a total sample size of *N* = 4000 was drawn. Prior to the draw, populations within previously drawn cities and villages were ordered in order of birth date. Then individual people were selected using the systematic random draw method. The study group included people without diagnosed kidney diseases (dialysis) or neoplastic diseases, non-pregnant and lactating women, and people who consciously consented to participate in the study. People on dialysis and those with neoplastic diseases were excluded because in these cases subjective taste impairment is possible, and alterations in taste sensitivity influence food preferences and appetite [30,31]. As a result, the study sample consisted of 1017 participants. 

### 2.3. Frequency of Eating Habits

A Dietary Habits and Nutrition Beliefs Questionnaire (KomPAN) [32,33] was used to assess the frequency of consumption of 11 food groups representing discretionary foods (refined plain bread; sausages; butter; margarine; lard and bacon; instant soups and ready-made soups; fast food; sweets; energy drinks; sweetened beverages) and 3 alcohol beverages (beer, wine and vodka).

All participants were asked to record their habitual frequency of consumption for each food group within the last three months according to the following categories: 1—less than once a month or never; 2—1–3 times a month; 3—once a week; 4—a few times a week; 5—once a day; 6—a few times a day. 

### 2.4. Perceived Health and Nutrition Concerns

The original Health Concern Scale (HCS) [34] was expanded by adding two statements on the concern about the risk of cancer and diabetes. A 7-point Likert scale starting from “definitely not” (1) through “neither no nor yes” (4) to “definitely yes” (7) was used to estimate each statement separately. Two indices were developed using the modified HCS.

The Health Concerns Index (HCI) was developed by use of five statements: (1) I am concerned about gaining weight; (2) I am concerned about the risk of high blood pressure; (3) I am concerned about the risk of coronary heart disease; (4) I am concerned about the risk of diabetes; and (5) I am concerned about the risk of cancer. 

The Nutrition Concern Index (NCI) included seven statements: (1) I am concerned about consuming many calories; (2) I am concerned about consuming a lot of fat in my food; (3) I am concerned about consuming a lot of cholesterol in my food; (4) I am concerned about consuming a lot of sugar in my food; (5) I am concerned about consuming a lot of salt in my food; (6) I am concerned about providing a sufficient amount of energy with my food; and (7) I am concerned about food additives in my food. 

The sum of the scores for each index was calculated. The range for HCI was 5–35 points and for NCI, 7—48 points. Participants were divided into three groups based on tertile distribution of HCI and NCI, separately.

### 2.5. Socio-Demographic Variables

Detailed sociodemographic data were collected: gender, age, education, place of residence. Data about weight and height were self-reported by the participants. Body mass index (BMI), adapting the Quetelet equation (body mass (kg)/height^2^ (m^2^)) was calculated and interpreted according to the criteria of the World Health Organization [35]. BMI 18.5–24.9 kg/m^2^ was assessed as normal, BMI 25.0–29.9 kg/m^2^ was considered overweight and BMI ≥ 30.0 kg/m^2^ obesity, while BMI < 18.5 kg/m^2^ was assessed as underweight [36].

### 2.6. Statistical Analysis

The obtained data are presented as means ± standard deviation (SD) with minimum, maximum, and median values. The distributions of the analyzed variables were verified with the Shapiro-Wilk test. To analyze the differences in NCI and HCI between two or more independent groups, the Mann-Whitney *U* test or Kruskal-Wallis analysis of variance (ANOVA) with Dunn’s post-hoc method with the Bonferroni correction were used, respectively. The accepted level of significance was set at *p* < 0.05. 

A principal components factor analysis (PCA) was conducted to derive dietary patterns based on the frequency of the consumption of ten food groups and three groups of alcohol (Table 1). The factors were rotated by an orthogonal (Varimax) transformation. The number of factors was based on the following criteria: components with an eigenvalue of 0.1, scree plot test, and the interpretability of the factors. The eigenvalues signify the amount of variance explained by each of the factors. Food items were considered to load on a factor if they had an absolute correlation 0.5 with it. A data-driven (a posteriori) approach was used to identify dietary patterns [37]. PDs were derived by principal component analyses (PCA) to which variables describing the frequency of eating some foods were introduced. The factorability of data was confirmed with the Kaiser-Meyer-Olkin (KMO) measure of sampling adequacy and Bartlett’s test of sphericity achieving statistical significance. The KMO value was 0.850, which attests the correct choice of analysis and the number of factors. Bartlett’s test had a significance of *p* < 0.0001 [38]. Four unhealthy dietary patterns (factors) were derived: “Fast food & alcohol” (factor 1), “Refined food & sweetened beverages” (factor 2), “Fat food” (factor 3), and “Butter” (factor 4) (Table 1). They explained 60.6% of total variance. 

Based on the tertiles distribution, participants were divided into three categories within each dietary pattern (bottom, middle, upper tertile). The upper tertile represents the greatest adherence and the bottom tertile represents the lowest adherence to the DP. 

Logistic regression analysis was used to verify associations between variables describing health and nutrition concerns (HCI, NCI), BMI and age (continuous ones), gender and education (categorical ones) as independent variables and DPs (dependent variables). 

Odds ratios (ORs) represent the chances of the adherence to upper tertiles of each DP. The reference groups (OR = 1.00) were those who represent the bottom tertile of each DP. Wald’s test was used to assess the significance of ORs [5]. *p*-value < 0.05 was considered as significant for all tests. All analyses were performed with IBM Statistics SPSS, version 26.0 (IBM Corp, Armonk, NY, USA).

## 3. Results

### 3.1. Sample Characteristics

The sample consisted of 1017 adults, including 623 women and 394 men. The socio-demographic characteristics of the study sample are presented in Table 2. Over 57% of the respondents had BMI > 25.0 kg/m^2^.

### 3.2. Health and Nutriton Concerns

Table 3 displays characteristics of the study sample in regards to health and nutrition concerns. The highest score was found for concern about the content of food additives in food (mean 4.1), while the lowest score was noted for concern about getting sufficient energy in one’s food (mean 3.5).

Higher concern about health was reported more often by women compared to men. Moreover, it was reported more by people over 50 compared to younger respondents, and also by those aged 36–50 compared to people aged 35 and younger. Higher health concerns were also reported by people living in cities of up to 100,000 inhabitants compared to those living in rural area, as well as overweight and obese people. Moreover, a higher concern for nutrition was shown by people over 50 years of age than by younger people, by overweight and obese people compared to others, by people living in cities, and by women. Education did not differentiate concern about health or about nutrition (Table 4).

### 3.3. Predictors of the Unhealthy Dietary Patterns 

The binary correlations between analyzed variables are presented in Table 5.

The results of logistic regression analysis are presented in Table 6. Women, people with higher education attainment, those living in large cities and younger people were more likely to consume foods from the “Fast food and alcohol” DP. Each subsequent year of life decreased the likelihood of adhering to this DP by 5%. People displaying higher NCI score and lower HCI score were more likely to often consume food included in the “Fast food & alcohol” DP. People with upper secondary education and lower education, those with higher BMI, and younger people were more likely to consume refined food, sweets and sweetened beverages (“Refined food & sweetened beverages” DP). Each subsequent year of life decreased the likelihood of being in the upper tertile of this DP by 3%. Each subsequent kg/m^2^ increased the likelihood of being in the upper tertile of this DP by 6%. Women and people displaying lower HCI score were less likely to consume refined food, sweets, and sweetened beverages. Women were also less likely to belong to the upper tertile of the “Fat food” DP compared to men. People with upper secondary education and lower education and those living in rural area were more likely to consume “fat foods”. Women were more likely to belong to the upper tertile of the “Butter” DP. However, people with primary and vocational education and those with higher BMI were less likely to consume butter. Each subsequent kg/m^2^ decreased the likelihood of often eating butter by 4%.

## 4. Discussion 

The purpose of the study was focused on the associations between perceived health and nutrition concerns, sociodemographic characteristics, and unhealthy dietary patterns. It was assumed that these factors may have different predictive importance in relation to unhealthy dietary behaviors. Our findings showed that sociodemographic characteristics differentiated both perceived health and nutrition concerns and adherence to unhealthy dietary patterns. Although the importance of sociodemographic features in differentiating individuals’ perceptions, attitudes and behaviors is still under discussion [39], our results justify including these features in the analysis. A previous study by Worsley and Lea [40] suggested that personal values may be stronger predictors of consumers’ concerns about food and health issues than demographics. However, in our study, some demographics turned out to discriminate individuals’ concerns. Women displayed health and nutrition concerns more than men, as confirmed by other studies [21,41]. Other research indicates that they are more conscious of body image and place higher importance on appearance compared to men [42,43]. More women than men perceive their image as overweight [41], and this may be conducive to their greater concerns for nutrition, especially in relation to the effect of food on body weight. Findings of our study confirmed that women were less likely to adhere to such unhealthy patterns as “Fast food & alcohol”, “Refined food & sweetened beverages”, and “Fat food” DPs. Thus, women show not only greater concern for health and nutrition but also have healthier dietary patterns than men. It may result from the greater awareness of women as they usually have more responsibility for complex decisions in everyday food preparation and consumption [44]. Therefore, men and women probably have different views on the risks related to food and nutrition. However, the cause-effect relationship between the reported concerns and behaviors is not obvious and this cross-sectional study does not allow deciding whether greater concerns for health and nutrition precede a more adequate diet, or the opposite. 

Findings of the study have also shown that health and nutrition concerns increased with BMI. Significantly higher health and nutrition concerns were also observed amongst young women who were overweight or obese [45]. Experienced health problems [46], dietary restrictions implemented by people with excess body weight [47], and repeated unsuccessful attempts at weight control may contribute to developing concern for health [48]. The associations between higher BMI and greater self-perceived risk of diseases such as cancer, heart diseases, and stroke were confirmed in previous studies [49]. There are relatively few studies showing a relationship between BMI and the perception of diet, i.e., concern about nutrition. It is known, however, that the healthy diet perception score may decrease with increasing BMI [50]. Higher diet quality may attenuate genetic predisposition to obesity, and therefore people with a lower BMI may perceive a healthy diet differently than those with a higher BMI [51]. The association between BMI and diet quality may lie deeper, being a result of participants’ social desirability bias. Thus, the relationship between being overweight or obese and the perceived concerns about health or nutrition still require further studying to understand some of the key issues in triggering behavioral change motivation.

At the same time, studies observe more frequent consumption of high-calorie food, as well as other habits leading to excess of energy supplied from the diet of the group of obese people [16,52,53]. In our study, people with a higher BMI were more likely to eat foods from the “Refined food & sweetened beverages” DP. Palatable foods high in fat and sugar are associated with increased food intake as they are more attractive and can be quickly converted into energy [54,55]. Additionally, it has been suggested that the high refined carbohydrate or fat content in this type of food may change the reward neurocircuitry, causing addictive-like eating behaviors and overconsumption [56,57]. Thus, the established dependence may be conditioned physiologically.

Similar to some studies [58,59] we did not find an association between the “Fast food and alcohol” DP and being overweight or obese. However, most of the previous studies indicated that BMI ≥ 25 kg/m^2^ is associated not only with a high intake of fast foods but also with high consumption of sugar-sweetened beverages [60]. The inconsistency in results may in part be explained by the cultural background; however, further research is still required to better understand this relationship.

The increase in concern for health and nutrition with age is noted in numerous studies [61,62] and also in our study. Age can correlate with an increase in the diet quality score among adults and the elderly, mainly due to the increased consumption of healthy food and the reduction of unhealthy food [7,63]. It appeared that adherence to “Fast food & alcohol” and “Refined food & sweetened beverages” DPs was related to age. Deteriorating health, but also the desire to maintain good health as long as possible, focuses people’s attention on health with age, including nutrition as a way to maintain health. This was confirmed by the decrease in the likelihood of adherence to “Fast food & alcohol” and “Refined food & sweetened beverages” DPs with age. Moreover, the relationship between some of the concerns (the concern about too many calories, concern about diseases) with age was confirmed by Sun [21].

The unexpected result was the lack of relationship between the education and both concerns about health and nutrition. However, education turned out to be a predictor of adherence to unhealthy DPs. People with an education lower than the upper secondary level were less likely to adhere to the “Fast food & alcohol” DP. At the same time, those people were more likely to adhere to the “Refined food & sweetened beverages” and “Fat food” DPs, which confirmed the results of other studies [8,64]. Generally, a low level of education determines the worse quality of the diet, which results from a large amount of fat and refined carbohydrates [65]. On the other hand, consumption of fast food by people with higher education may result from their greater social activity, including that associated with special occasions [66].

We confirmed that unhealthy dietary patterns are differentiated internally, and therefore, an individual’s characteristics, including demographics and those related to health and nutrition concerns, may have predictive importance. We hypothesized that respondents who were more concerned about health or nutrition would be less likely to display unhealthy dietary behaviors, and this has been partly confirmed. The study showed that higher levels of health concerns decreased the chances of more frequent consumption of foods characteristic for “Fast food & alcohol” and “Refined food & sweetened drinks” DPs. However, no relationship was found for other foods containing a lot of fat (“Fat food” and “Butter” DPs). Previous studies indicated positive relationship between health concerns and organic food consumption [67,68], as well as between health concerns and healthy dietary patterns [45]. It can be assumed that the perceived health-related concerns may therefore lead to the improvement of the diet by introducing healthy behaviors, but they are not sufficient to eliminate unfavorable behaviors. This can be explained by the stronger relationship between health concerns and the cognitive component of the attitude toward health, while unfavorable behaviors are related more to preferences and therefore reflect the affective component rather than the cognitive component [69,70]. Lack of positive relationship between health concerns and the consumption of high-fat foods can suggest an optimistic bias, where the individual underestimates his/her susceptibility to controllable diseases. An alternative explanation is the lack of knowledge about health risk factors, including fat, but this is unlikely due to increasing nutritional awareness [71].

Higher level of concerns about nutrition lowered the likelihood of consuming refined food and sweetened beverages, however it increased the chances of more frequent consumption of foods characteristic for “Fast food & alcohol” DPs. Such association between consumption of fatty foods and concerns about nutrition is confirmed also in studies by Noureddine and Metzger [72]. This may result from the fact that the individual’s decisions related to the choice of food are influenced not only by their characteristics (including the perceived concern for health) but also by the environment [73]. Hence, people who perceive potential health risks may be strongly influenced by the environment (e.g., the tradition of eating certain food), which makes it difficult to change behavior regarding healthy eating. Thus, healthy behavior will not be observed. Fast food eaten as a snack helps people cope with negative emotions or is a component of enjoying a special occasion and is a way of gaining “fast” energy [66]. When faced with health problems, these psychological and social motives can be overwhelming and lead to eating unhealthy foods. In addition, taste preferences can play an important role throughout life and determine food choices [74]. Moreover, people do not necessarily associate their concerns about health with their eating behaviors [72], which suggests that trying to motivate people to eat healthy by focusing on their health concerns may not be an effective strategy. Because individual food choices are influenced by the environment, they are impacted by the comprehensive approach to population health, sustainability and legislation. Therefore, strategies which cover health care, food markets, information management and education while taking into account cultural, social and economic context [73] can significantly help individuals in managing their consumption of unhealthy food and their concerns related to it.

### Strengths and Limitations

The strength of our results is a relatively large representative sample of Polish adults, which allows for high confidence in the study results. It is a national population-based sample, with a valid selection of the study group, carried out by professional interviewers. However, our findings are specific to the Polish cultural background and should be used with caution in relation to other populations. Moreover, there are several limitations regarding the study, which should be considered. One of them relates to the potential biases that may occur when self-reported data are analyzed. When providing self-reported weight and height, men typically overestimate height and women underestimate weight [75], which may also affect our results. Self-reported data was collected as it was logistically impossible for the researchers to make measurements, taking into account sample size. The use of food frequency to drive dietary patterns is also a limitation due to overestimation of consumption of some foods when frequency of eating is measured [76]. However, we have chosen questionnaire KomPAN [33] because we aimed to see predominantly “unhealthy” dietary patterns rather than the exact amount of foods. It should be also stated that this was a cross-sectional study and did not allow us to assess the causality of relationships between the variables. Although findings should not be generalized to a population with a different cultural background, our study provides an interesting insight into internal differences within unhealthy dietary patterns and their associations with sociodemographic characteristics, as well as perceived health and nutrition concerns. 

## 5. Conclusions

Our study showed that the relationship between perceived concerns about health and nutrition and frequency of consumption of unhealthy food did not confirm our assumptions. Higher levels of health concerns decreased the chances of adhering to upper tertiles of “Fast food & alcohol” and “Refined food & sweetened beverages” DPs, thus displaying less frequent consumption of foods characteristic for these patterns. However, no relationship was found between health concerns and frequency of consuming other foods containing a lot of fat (“Fat food” and “Butter” DPs). Nutrition concerns increased the likelihood of frequent consumption of foods from the “Fast food & alcohol” DP. It appeared that women were less likely to adhere to unhealthy eating patterns than men, while older people were less likely to often consume fast food, alcohol, refined and sweetened beverages compared to younger respondents. Findings of this study showed the different nature of the relationships between the consumption of unhealthy food and concerns about health compared to the relationships between the consumption of unhealthy food and the concern about nutrition. These relationships should be considered when developing interventions to address health-related lifestyle changes. However, further research is needed to identify cause-effect relationships between these variables.

Determining the characteristics of unhealthy dietary patterns among Poles and exploring the health and nutrition concerns that coexist with those patterns is important for increasing the efficiency of health interventions. Unhealthy eating patterns can be modified through a variety of policies (ranging from health education programs to targeted pricing and regulatory interventions related to certain harmful or beneficial components of the diet) due to the inverse relationship between perceived dietary concerns and the consumption of unhealthy food. Moreover, efforts are needed to improve public health messages about how lifestyle risk factors impact the chances of developing diseases. These messages need to be adapted to the sociodemographic specificity of risk groups. The results obtained can be used in developing the interventions aimed at lifestyle changes related to health while focusing on the perceived concern for health and nutrition and their relationship with the consumption of unhealthy food.

## Figures and Tables

**Table 1:** Factor-loading matrix for the dietary patterns DPs identified by principal component analysis (PCA; correlation coefficients).

Food Groups and Alcohol	Factor 1	Factor 2	Factor 3	Factor 4
White bread and bakery products, e.g., wheat bread, rye bread, wheat/rye bread, toast bread, bread rolls	−0.221	0.726 *	0.246	0.095
Cold meats, smoked sausages, hot-dogs	−0.093	0.481	0.601 *	0.089
Butter as a bread spread or as an addition to meals/for frying/for baking	−0.158	0.153	0.152	0.936 *
Lard as a bread spread, or as an addition to meals/for frying/for baking etc.	0.239	0.069	0.738 *	−0.041
Instant soups or ready-made soups, e.g., tinned, jar, concentrates (excluding frozen soup mixes)	0.610 *	−0.088	0.414	0.059
Tinned (jar) meats	0.471	−0.081	0.626 *	0.087
Fast foods, e.g., potato chips, hamburgers, pizza, hot-dogs	0.705 *	0.071	0.209	0.035
Sweets, e.g., confectionary, biscuits, cakes, chocolate bars, cereal bars, other	0.238	0.611 *	−0.142	−0.142
Energy drinks such as Red Bull, Monster, Rockstar or other	0.768 *	0.131	0.056	0.069
Sweetened carbonated or still beverages such as Coca-Cola, Pepsi, Sprite, Fanta, lemonade	0.495	0.608 *	0.021	0.095
Beer	0.644 *	0.275	0.136	0.037
Wine	0.701 *	−0.139	0.029	−0.061
Vodka	0.744 *	0.108	0.106	−0.035
Variance Explained (%)	30.21	11.4	11.3	7.8
Total Variance Explained (%)	60.6			

* Correlations coefficients higher than 0.6.

**Table 2:** Study sample characteristics.

Variables		*N* = 1017	%
Gender	Women	623	61.3
	Men	394	38.7
Education	Primary	130	12.8
	Vocational	304	29.8
	Upper secondary	443	43.6
	Higher	140	13.8
Place of residence	Rural area	493	48.5
	City ≤ 100,000 residents	306	30.1
	City > 100,000 residents	218	21.4
Age (years)	≤35	281	27.6
	36–50	240	23.7
	51–65	273	26.8
	>65	223	21.9
Age in years (mean ± standard deviation)		49.3 ± 17.7	
BMI category (kg/m^2^)	Underweight (<18.5)	19	1.9
	Normal weight (18.5–24.9)	413	40.6
	Overweight (25.0–29.9)	429	42.2
	Obesity (≥30)	156	15.3
BMI in kg/m^2^ (mean ± standard deviation)		25.9 ± 4.3	

*N*—number of participants.

**Table 3:** Perceived health and nutrition concerns (mean ± standard deviation, median, minimum–maximum) *.

Indices/Statements	Mean *; Standard Deviation	Median (Minimum–Maximum)
**Health Concern Index (HCI)**	19.7 ± 6.41	20 ** (5–35)
(1) I am concerned about gaining weight	3.9 ± 1.65	4 ** (1–7)
(2) I am concerned about the risk of high blood pressure	4.1 ± 1.57	4 ** (1–7)
(3) I am concerned about the risk of coronary heart disease	3.9 ± 1.52	4 ** (1–7)
(4) I am concerned about the risk of diabetes	3.9 ± 1.58	4 ** (1–7)
(5) I am concerned about the risk of cancer	4.0 ± 1.55	4 ** (1–7)
**Nutrition Health Concern (NCI)**	26.5 ± 6.56	27 ** (7–48)
(1) I am concerned about consuming many calories	3.5 ± 1.50	3 ** (1–7)
(2) I am concerned about consuming a lot of fat in my food	3.8 ± 1.52	4 ** (1–7)
(3) I am concerned about consuming a lot of cholesterol in my food	3.9 ± 1.56	4 ** (1–7)
(4) I am concerned about consuming a lot of sugar in my food	3.8 ± 1.59	4 ** (1–7)
(5) I am not concerned about consuming a lot of salt in my food	3.8 ± 1.56	4 ** (1–7)
(6) I am concerned about my food providing a sufficient amount of energy	3.5 ± 1.54	3 ** (1–7)
(7) I am concerned about food additives in my food	4.1 ± 1.55	4 ** (1–7)

* a 7-point scale starting from “definitely not” (1) through “neither no nor yes” (4) to “definitely yes” (7). ** distribution different than normal (verified using Shapiro-Wilk test—*p* < 0.05).

**Table 4:** Perceived health and nutrition concerns in the study sample (*N* = 1017).

Variable		Concerns about:			
		Health (HCI) (Range: 5–35 Points)		Nutrition (NCI) (Range: 7–48 Points)	
		Mean ± SD	*p*-Value	Mean ± SD	*p*-Value
Gender *	Female	20.3 ± 6.42	<0.001	27.3 ± 8.60	<0.001
	Male	18.8 ± 6.31		25.2 ± 8.37	
Age (years) **	≤35	17.9 ^a^ ± 6.68	<0.001	25.1 ^a^ ± 8.88	0.002
	36–50	19.2 ^b^ ± 6.19		26.1 ^ad^ ± 8.27	
	51–65	20.7 ^c^ ± 6.14		27.6 ^bc^ ± 8.54	
	>65	21.4 ^c^ ± 6.00		27.3 ^cd^ ± 8.26	
Place of residence **	Rural area	19.2 ^a^ ± 6.25	0.032	25.7 ^a^ ± 8.55	0.004
	City ≤ 100,000 residents	20.5 ^b^ ± 6.57		27.8 ^b^ ± 8.49	
	City > 100,000 residents	19.7 ^ab^ ± 6.50		26.4 ^ab^ ± 8.51	
Education **	Primary	20.4 ± 6.94	0.553	26.3 ± 8.94	0.087
	Vocational	19.4 ± 6.40		25.5 ± 8.71	
	Upper secondary	19.6 ± 6.37		27.1 ± 8.55	
	Higher	19.9 ± 6.10		26.7 ± 7.78	
BMI category (kg/m^2^) **	Underweight (<18.5)	14.6 ^a^ ± 6.18	<0.001	21.6 ^a^ ± 9.03	<0.001
	Normal weight (18.5–24.9)	18.1 ^b^ ± 6.44		25.3 ^a^ ± 8.66	
	Overweight (25.0–29.9)	20.9 ^c^ ± 6.24		27.6 ^b^ ± 8.53	
	Obesity (≥30)	21.4 ^c^ ± 5.58		26.9 ^b^ ± 7.79	

SD—standard deviation. * U Mann-Whitney test ** Kruskall-Wallis with Dunn’s post-hoc test. ^abcd^ values with different letter differed significantly at *p* < 0.05.

**Table 5:** Bivariate correlations between variables.

Number	Variable	Number of Variable										
		1.	2.	3.	4.	5.	6.	7.	8.	9.	10.	11.
1.	Gender	1										
2.	Education	−0.135 **	1									
3.	Place of residence	−0.056	0.231 **	1								
4.	Age	−0.029	−0.410 **	0.040	1							
5.	BMI	0.158 **	−0.268 **	−0.057	0.383 **	1						
6.	Health concern	−0.112 **	−0.010	0.043	0.223 **	0.240 **	1					
7.	Nutrition concern	−0.116 **	0.049	0.054	0.120 **	0.113 **	0.818 **	1				
8.	Factor 1 ***	0.126 **	0.186 **	0.047	−0.364 **	−0.125 **	−0.120 **	−0.025	1			
9.	Factor 2	0.175 **	−0.142 **	−0.101 **	−0.095 **	0.056	−0.175 **	−0.192 **	0.000	1		
10.	Factor 3	0.045	−0.157 **	−0.098 **	0.084 **	0.041	0.055	−0.018	0.000	0.000	1	
11.	Factor 4	−0.131 *	0.138 **	−0.008	−0.005	−0.084 *	−0.018	−0.019	0.000	0.000	0.000	1

* correlation significant at *p* = 0.05; ** correlation significant at *p* = 0.01. *** Factor 1—“Fast food & alcohol” DP; Factor 2—“Refined food & sweetened beverages” DP; Factor 3—“Fat food” DP; Factor 4—“Butter” DP.

**Table 6:** Associations between dietary patterns and selected characteristics of the study sample (Odds Ratios with 95% Confidence Intervals).

Variables	“Fast Food & Alcohol” DP (Factor 1)			“Refined Food & Sweetened Beverages” DP (Factor 2)			“Fat Food” DP (Factor 3)			“Butter” DP (Factor 4)		
	Bottom Tertile	Middle Tertile	Upper Tertile	Bottom Tertile	Middle Tertile	Upper Tertile	Bottom Tertile	Middle Tertile	Upper Tertile	Bottom Tertile	Middle Tertile	Upper Tertile
Gender												
Female	1	0.54 *** (0.38; 0.77)	0.39 **** (0.27; 0.57)	1	0.84 (0.60; 1.18)	0.54 **** (0.38; 0.75)	1	0.92 (0.66; 1.27)	0.71 * (0.51; 0.99)	1	1.08 (0.88; 1.47)	1.80 *** (1.31; 2.47)
Male (ref.)	1	1	1	1	1	1	1	1	1	1	1	1
Education												
Primary	1	0.33 *** (0.17; 0.66)	0.36 ** (0.17; 0.76)	1	3.77 **** (1.93; 7.32)	4.62 **** (2.29; 9.32)	1	2.35 ** (1.23; 4.52)	3.37 **** (1.72; 6.58)	1	0.41 ** (0.22; 0.76)	0.56 * (0.31; 0.99)
Vocational	1	0.56 * (0.32; 0.98)	0.64 (0.35; 1.15)	1	2.51 **** (1.46; 4.29)	3.36 **** (1.94; 5.84)	1	3.02 **** (1.80; 5.06)	3.39 **** (1.94; 5.92)	1	0.53 * (0.32; 0.88)	0.43 ** (0.26; 0.71)
Upper secondary	1	0.96 (0.57; 1.63)	1.10 (0.64; 1.90)	1	2.02 ** (1.26; 3.24)	1.68 * (1.03; 2.73)	1	1.48 (0.94; 2.34)	2.10 ** (1.28; 3.45)	1	1.01 (0.62; 1.65)	0.84 (0.52; 1.36)
Higher (ref.)	1	1	1	1	1	1	1	1	1	1	1	1
Place of residence												
Rural area	1	0.98 (0.63; 1.52)	0.62 * (0.39; 0.97)	1	1.41 (0.94; 2.13)	1.15 (0.75; 1.75)	1	1.34 (0.90; 2.00)	1.68 * (1.11; 2.55)	1	1.03 (0.70; 1.52)	1.10 (0.74; 1,63)
City ≤ 100,000	1	1.06 (0.66; 1.69)	0.67 (0.42; 1.09)	1	0.97 (0.63; 1.50)	0.90 (0.58; 1.40)	1	1.39 (0.91; 2.12)	1.44 (0.92; 2.24)	1	1.03 (0.67; 1.57)	1.26 (0.82; 1.92)
City > 100,000 (ref.)	1	1	1	1	1	1	1	1	1	1	1	1
Age (years)	1	0.97 **** (0.96; 0.98)	0.95 **** (0.94; 0.96)	1	0.99 (0.98; 1.00)	0.97 **** (0.96; 0.98)	1	1.00 (0.99; 1.01	1.01 (0.99; 1.02)	1	0.99 (0.98; 1.01)	1.00 (0.99; 1.01)
BMI (kg/m^2^)	1	0.99 (0.95; 1.03)	0.98 (0.93; 1.02)	1	1.00 (0.96; 1.04)	1.06 ** (1.02; 1.11)	1	1.01 (0.97; 1.05)	1.00 (0.96; 1.04)	1	0.95 ** (0.91; 1.00)	0.96 * (0.93; 0.99)
Health Concern Index (HCI)	1	0.97 (0.93; 1.01)	0.93 ** (0.89; 0.98)	1	1.03 (0.99; 1.08)	0.95 * (0.91; 1.00)	1	1.01 (0.97; 1.06)	1.02 (0.98; 1.07)	1	1.00 (0.98; 1.03)	0.99 (0.97; 1.01)
Nutrition Concern Index (NCI)	1	1.03 (0.99; 1.06)	1.07 **** (1.03; 1.11)	1	0.96 * (0.93; 0.99)	0.98 (0.95; 1.01)	1	0.99 (0.96; 1.03)	0.99 (0.96; 1.02)	1	1.00 (0.99; 1.02)	0.99 (0.97; 1.00)

* *p* < 0.05; ** *p* < 0.01; *** *p* < 0.001; **** *p* < 0.0001 (Wald’s test).

## Data Availability

The data presented in this study are available on request from the corresponding author.

## References

[1] Mueller N.T., Appel L.J. (2017). Attributing death to diet: Precision counts. JAMA.

[2] Khandelwal S., Kurpad A., Narayan K.M.V. (2018). Global Non-Communicable Diseases—The Nutrition Conundrum. Front. Public Health.

[3] WHO (2014). Global Status Report on Non-Communicable Diseases. http://www.who.int/global-coordination-mechanism/publications/global-status-report-ncds-2014-eng.pdf.

[4] Kvaavik E., Batty G., Ursin G., Huxley R., Gale C.R. (2010). Influence of individual and combined health behaviors on total and cause-specific mortality in men and women: The United Kingdom health and lifestyle survey. Arch. Intern. Med..

[5] Loef M., Walach H. (2012). The combined effects of healthy lifestyle behaviors on all-cause mortality: A systematic review and meta-analysis. Prev. Med..

[6] WHO (2018). World Health Organization. Obesity and Overweight Factsheet..

[7] Hu E.A., Toledo E., Diez-Espino J., Estruch R., Corella D., Salas-Salvado J., Vinyoles E., Gomez-Gracia E., Aros F., Fiol M. (2013). Lifestyles and Risk Factors Associated with Adherence to the Mediterranean Diet: A Baseline Assessment of the PREDIMED Trial. PLoS ONE.

[8] Thorpe M.G., Milte C.M., Crawford D., McNaughton S.A. (2019). Education and lifestyle predict change in dietary patterns and diet quality of adults 55 years and over. Nutr. J..

[9] Cespedes E.M., Hu F.B. (2015). Dietary patterns: From nutritional epidemiologic analysis to national guidelines. Am. J. Clin. Nutr..

[10] Reedy J., Krebs-Smith S.M., Hammond R.A., Hennessy E. (2017). Advancing the science of dietary patterns research to leverage a complex systems approach. J. Acad. Nutr. Diet..

[11] Sui Z., Wong W.K., Louie J.C., Rangan A. (2017). Discretionary food and beverage consumption and its association with demographic characteristics, weight status, and fruit and vegetable intakes in Australian adults. Public Health Nutr..

[12] Schmid A., Gille D., Piccinali P., Bütikofer U., Chollet M., Altintzoglou T., Honkanen P., Walther B., Stoffers H. (2017). Factors predicting meat and meat products consumption among middle-aged and elderly people: Evidence from a consumer survey in Switzerland. Food Nutr. Res..

[13] Waśkiewicz A., Szcześniewska D., Szostak-Węgierek D., Kwaśniewska M., Pająk A., Stepaniak U., Kozakiewicz K., Tykarski A., Zdrojewski T., Zujko M.E. (2016). Are dietary habits of the Polish population consistent with the recommendations for prevention of cardiovascular disease? WOBASZ II project. Kardiol. Pol..

[14] Eilander A., Harika R.K., Zock P.L. (2015). Intake and sources of dietary fatty acids in Europe: Are current population intakes of fats aligned with dietary recommendations?. Eur. J. Lipid Sci. Technol..

[15] Azaïs-Braesco V., Sluik D., Maillot M., Kok F., Moreno L.A. (2017). A review of total & added sugar intakes and dietary sources in Europe. Nutr. J..

[16] Johnson B.J., Bell L.K., Zarnowiecki D., Rangan A.M., Golley R.K. (2017). Contribution of Discretionary Foods and Drinks to Australian Children’s Intake of Energy, Saturated Fat, Added Sugars and Salt. Children.

[17] Mohammadbeigi A., Asgarian A., Moshir E., Heidari H., Afrashteh S., Khazaei S., Ansari H. (2018). Fast food consumption and overweight/obesity prevalence in students and its association with general and abdominal obesity. J. Prev. Med. Hyg..

[18] Rauber F., Louzada M.L.D.C., Martinez Steele E., Rezende L.F.M., Millett C., Monteiro C.A., Levy R.B. (2019). Ultra-processed foods and excessive free sugar intake in the UK: A nationally representative cross-sectional study. BMJ Open.

[19] Van Bussel B.C., Soedamah-Muthu S.S., Henry R.M., Schalkwijk C.G., Ferreira I., Chaturvedi N., Toeller M., Fuller J.H., Stehouwer C.D., EURODIAB Prospective Complications Study Group (2013). Unhealthy dietary patterns associated with inflammation and endothelial dysfunction in type 1 diabetes: The EURODIAB study. Nutr. Metab. Cardiovasc. Dis..

[20] Roininen K., Lähteenmäki L., Tuorila H. (1999). Quantification of consumer attitudes to health and hedonic characteristics of foods. Appetite.

[21] Sun Y.-H.C. (2008). Health concern, food choice motives, and attitudes toward healthy eating: The mediating role of food choice motives. Appetite.

[22] Tromp D.M., Brouha X.D.R., Hordijk G.J., Winnubst J.A.M., Gebhardt W.A., van der Doef M.P., de Leeuw J.R. (2005). Medical care seeking and health-risk behaviour in patients with head and neck cancer: The role of health value, control beliefs and psychological distress. Health Educ. Res..

[23] Leikas S., Lindeman M., Roininen K., Lähteenmäki L. (2009). Who is responsible for food risks? The influence of risk type and risk characteristics. Appetite.

[24] Imes C.C., Lewis F.M. (2014). Family history of cardiovascular disease, perceived cardiovascular disease risk, and health-related behavior: A review of the literature. J. Cardiovasc. Nurs..

[25] Acheson L.S., Wang C., Zyzanski S.J., Lynn A., Ruffin M.T., Gramling R., Rubinstein W.S., O’Neill S.M., Nease D.E. (2010). Family history and perceptions about risk and prevention for chronic diseases in primary care: A report from the family healthcare impact trial. Genet. Med..

[26] McCusker M.E., Yoon P.W., Gwinn M., Malarcher A.M., Neff L., Khoury M.J. (2004). Family history of heart disease and cardiovascular disease risk-reducing behaviors. Genet. Med..

[27] Andersson P., Sjoberg R.L., Ohrvik J., Leppert J. (2009). The effects of family history and personal experiences of illness on the inclination to change health-related behaviour. Cent. Eur. J. Public Health.

[28] Krieger J.P., Pestoni G., Cabaset S., Brombach C., Sych J., Schader C., Faeh D., Rohrmann S. (2018). Dietary Patterns and Their Sociodemographic and Lifestyle Determinants in Switzerland: Results from the National Nutrition Survey *menuCH*. Nutrients.

[29] Martinez-Lacoba R., Pardo-Garcia I., Amo-Saus E., Escribano-Sotos F. (2020). Social determinants of food group consumption based on Mediterranean diet pyramid: A cross-sectional study of university students. PLoS ONE.

[30] Fitzgerald C., Wiese G., Moorthi R.N., Moe S.M., Hill Gallant K., Running C.A. (2019). Characterizing Dysgeusia in Hemodialysis Patients. Chem. Senses.

[31] Pugnaloni S., Vignini A., Borroni F., Sabbatinelli J., Alia S., Fabri M., Taus M., Mazzanti L., Berardi R. (2020). Modifications of taste sensitivity in cancer patients: A method for the evaluations of dysgeusia. Support Care Cancer.

[32] (2014). Kwestionariusz do Badania Poglądów i Zwyczajów Żywieniowych Oraz Procedura Opracowania Danych (KomPAN^®^): Wersja Polskojęzyczna [Dietary Habits and Nutrition Beliefs Questionnaire and the Manual for Developing of Nutritional Data (KomPAN]. Committee of Human Nutrition Science. Polish Academy of Science. Warsaw. http://www.knozc.pan.pl/.

[33] Kowalkowska J., Wadolowska L., Czarnocinska J., Człapka-Matyasik M., Galiński G., Jezewska-Zychowicz M., Bronkowska M., Dlugosz A., Loboda D., Wyka J. (2018). Reproducibility of a Questionnaire for Dietary Habits, Lifestyle and Nutrition Knowledge Assessment (KomPAN^®^) in Polish Adolescents and Adults. Nutrients.

[34] Kähkönen P., Tuorila H. (1999). Consumer responses to reduced and regular fat content in different products: Effects of gender, involvement and health concern. Food Qual. Prefer..

[35] Branca F., Nikogosian H., Lobstein T., WHO (2007). The Challenge of Obesity in the WHO European Region and the Strategies for Response: Summary.

[36] Ashwell M., Gibson S. (2016). Waist-to-height ratio as an indicator of ‘early health risk’: Simpler and more predictive than using a ‘matrix’ based on BMI and waist circumference. BMJ Open.

[37] Newby P.K., Tucker K.L. (2004). Empirically derived eating patterns using factor or cluster analysis: A review. Nutr. Rev..

[38] Wirfält E., Drake I., Wallström P. (2013). What do review papers conclude about food and dietary patterns?. Food Nutr. Res..

[39] McGowan L., Pot G.K., Stephen A.M., Lavelle F., Spence M., Raats M., Hollywood L., McDowell D., McCloat A., Mooney E. (2016). The influence of socio-demographic, psychological and knowledge-related variables alongside perceived cooking and food skills abilities in the prediction of diet quality in adults: A nationally representative cross-sectional study. Int. J. Behav. Nutr. Phys. Act..

[40] Worsley A., Lea E. (2008). Consumer concerns about food and health: Examination of general and specific relationships with personal values and demographics. Br. Food J..

[41] Chung W.-S., Shin K.O., Bae J.Y. (2019). Gender Differences in Body Image Misperception According to Body Mass Index, Physical Activity, and Health Concern among Korean University Students. J. Men Health.

[42] Salameh P., Jomaa L., Issa C., Farhat G., Salamé J., Zeidan N., Baldi I. (2014). Assessment of Dietary Intake Patterns and Their Correlates among University Students in Lebanon. Front. Public Health.

[43] Penney T.L., Kirk S.F.L. (2015). The health at every size paradigm and obesity: Missing empirical evidence may help push the reframing obesity debate forward. Am. J. Public Health.

[44] Grundtvig S. (2006). Savoury Dishes for Adult Education and Counselling: Food Literacy Guidelines and Toolbox.

[45] Jezewska-Zychowicz M., Wadolowska L., Kowalkowska J., Lonnie M., Czarnocinska J., Babicz-Zielińska E. (2017). Perceived Health and Nutrition concerns as predictors of dietary patterns among Polish Females aged 13-21 years (GEBaHealth Project). Nutrients.

[46] Luppino F.S., de Wit L.M., Bouvy P.F., Stijnen T., Cuijpers P., Penninx B.W., Zitman F.G. (2010). Overweight, obesity, and depression: A systematic review and meta-analysis of longitudinal studies. Arch. Gen. Psychiatry.

[47] Price M., Higgs S., Lee M. (2015). Self-reported eating traits: Underlying components of food responsivity and dietary restriction are positively related to BMI. Appetite.

[48] Leme A.C.B., Lubans D.R., Guerra P.H., Dewar D., Toassa E.C., Philippi S.T. (2016). Preventing obesity among Brazilian adolescent girls: Six-month outcomes of the Healthy Habits, Healthy Girls—Brazil school-based randomized controlled trial. Prev. Med..

[49] Finkelstein E.A., Brown D.S., Evans W.D. (2008). Do obese persons comprehend their personal health risks?. Am. J. Health Behav..

[50] Ferrão A.C., Guiné R.P.F., Correia P., Ferreira M., Cardoso A.P., Duarte J., Lima J. (2018). Perceptions towards a healthy diet among a sample of university people in Portugal. Nutr. Food Sci..

[51] Ding M., Ellervik C., Huang T., Jensen M.K., Curhan G.C., Pasquale L.R., Kang J.H., Wiggs J.L., Hunter D.J., Willett W.C. (2018). Diet quality and genetic association with body mass index: Results from 3 observational studies. Am. J. Clin. Nutr..

[52] Wright S.M., Aronne L.J. (2012). Causes of obesity. Abdom. Imaging.

[53] Poti J.M., Braga B., Qin B. (2017). Ultra-processed Food Intake and Obesity: What Really Matters for Health-Processing or Nutrient Content?. Curr. Obes. Rep..

[54] Estadella D., Oyama L.M., Dâmaso A.R., Ribeiro E.B., Do Nascimento C.M.O. (2004). Effect of palatable hyperlipidic diet on lipid metabolism of sedentary and exercised rats. Nutrition.

[55] Erlanson-Albertsson C. (2005). How palatable food disrupts appetite regulation. Basic Clin. Pharmacol. Toxicol..

[56] Schulte E.M., Avena N.M., Gearhardt A.N. (2015). Which foods may be addictive? The roles of processing, fat content, and glycemic load. PLoS ONE.

[57] Carter A., Hendrikse J., Lee N., Yucel M., Verdejo-Garcia A., Andrews Z., Hall W. (2016). The Neurobiology of “Food Addiction” and Its Implications for Obesity Treatment and Policy. Annu. Rev. Nutr..

[58] Barnes T.L., French S.A., Mitchell N.R., Wolfson J. (2016). Fast-food consumption, diet quality and body weight: Cross-sectional and prospective associations in a community sample of working adults. Public Health Nutr..

[59] Nurwanti E., Uddin M., Chang J.-S., Hadi H., Syed-Abdul S., Su E., Nursetyo A., Masud J., Bai C.-H. (2018). Roles of sedentary behaviors and unhealthy foods in increasing the obesity risk in adult men and women: A cross-sectional national study. Nutrients.

[60] Kaiser K.A., Brown A.W., Bohan Brown M.M., Shikany J.M., Mattes R.D., Allison D.B. (2014). Increased fruit and vegetable intake has no discernible effect on weight loss: A systematic review and meta-analysis. Am. J. Clin. Nutr..

[61] Laditka J.N., Laditka S.B., Liu R., Price A.E., Wu B., Friedman D.B., Corwin S.J., Sharkey J.R., Tseng W., Hunter R. (2011). Older adults’ concerns about cognitive health: Commonalities and differences among six United States ethnic groups. Ageing Soc..

[62] Faller J.W., Marcon S.S. (2013). Health care and socio-cultural practices for elderly patients in different ethnic groups. Esc. Anna Nery..

[63] De Andrade S.C., Previdelli Á.N., Cesar C.L., Marchioni D.M., Fisberg R.M. (2016). Trends in diet quality among adolescents, adults and older adults: A population-based study. Prev. Med. Rep..

[64] Bolt-Evensen K., Vik F.N., Stea T.H., Klepp K.I., Bere E. (2018). Consumption of sugar-sweetened beverages and artificially sweetened beverages from childhood to adulthood in relation to socioeconomic status—15 years follow-up in Norway. Int. J. Behav. Nutr. Phys. Act..

[65] López-Olmedo N., Popkin B., Taillie L.S. (2019). Association between socioeconomic status and diet quality in Mexican men and women: A cross-sectional study. PLoS ONE.

[66] Verhoeven A.A., Adriaanse M.A., de Vet E., Fennis B.M., de Ridder D.T. (2015). It’s my party and I eat if I want to. Reasons for unhealthy snacking. Appetite.

[67] Magnusson M.K., Arvola A., Hursti U.K.K., Abergb L., Sjödén P.O. (2003). Choice of organic foods is related to perceived consequences for human health and to environmentally friendly behaviour. Appetite.

[68] Chen M. (2009). Attitude toward organic foods among Taiwanese as related to health consciousness, environmental attitudes, and the mediating effects of a healthy lifestyle. Br. Food J..

[69] Babicz-Zielińska E., Rybowska A., Zabrocki R. (2006). Relations between emotions and food preferences. Pol. J. Food Nutr. Sci..

[70] Wehling H., Lusher J.M. (2019). Cognitive and Emotional Influences on Eating Behaviour: A Qualitative Perspective. Nutr. Metab. Insights.

[71] Yahia N., Brown C.A., Rapley M., Chung M. (2016). Level of nutrition knowledge and its association with fat consumption among college students. BMC Public Health.

[72] Noureddine S., Metzger B. (2014). Do health-related feared possible selves motivate healthy eating?. Health Psychol. Res..

[73] Fanzo J., Davis C. (2019). Can Diets Be Healthy, Sustainable, and Equitable?. Curr. Obes. Rep..

[74] Liem D.G., Russell C.G. (2019). The Influence of Taste Liking on the Consumption of Nutrient Rich and Nutrient Poor Foods. Front. Nutr..

[75] Burton N.W., Brown W., Dobson A. (2010). Accuracy of body mass index estimated from self-reported height and weight in mid-aged Australian women. Aust. N. Z. J. Public Health.

[76] Kowalkowska J., Slowinska M.A., Slowinski D., Dlugosz A., Niedzwiedzka E., Wadolowska L. (2013). Comparison of a full food-frequency questionnaire with the three-day unweighted food records in young Polish adult women: Implications for dietary assessment. Nutrients.

